# Methylene blue in anticancer photodynamic therapy: systematic review of preclinical studies

**DOI:** 10.3389/fphar.2023.1264961

**Published:** 2023-09-28

**Authors:** Amir Taldaev, Roman Terekhov, Ilya Nikitin, Elizaveta Melnik, Vera Kuzina, Mikhail Klochko, Igor Reshetov, Artem Shiryaev, Victor Loschenov, Galina Ramenskaya

**Affiliations:** ^1^ Laboratory of Nanobiotechnology, Institute of Biomedical Chemistry, Moscow, Russia; ^2^ Phystech School of Biological and Medical Physics, Moscow Institute of Physics and Technology (State University), Dolgoprudny, Russia; ^3^ Nelyubin Institute of Pharmacy, I. M. Sechenov First Moscow State Medical University (Sechenov University), Moscow, Russia; ^4^ Department of Oncology, Radiotherapy and Reconstructive Surgery, University Clinical Hospital No. 1, Levshin Institute of Cluster Oncology, I. M. Sechenov First Moscow State Medical University (Sechenov University), Moscow, Russia; ^5^ Department of Laser Micro-Nano and Biotechnology, National Research Nuclear University MEPhI (Moscow Engineering Physics Institute), Moscow, Russia

**Keywords:** methylene blue, cancer, tumor, photodynamic therapy, nanopharmaceutics, systematic review

## Abstract

**Background:** Methylene blue has a long history of clinical application. Thanks to phenothiazine chromophore, it has potential in photodynamic anticancer therapy. In spite of the growing body of literature that has evaluated the action of this dye on different types of cancer, the systematic understanding of this problem is still lacking. Therefore, this systematic review was performed to study the efficacy of methylene blue in photodynamic anticancer therapy.

**Methods:** This systematic review was carried out in accordance with the PRISMA guidelines, and the study protocol was registered in PROSPERO (CRD42022368738). Articles for the systematic review were identified through the PubMed database. SYRCLE’s risk of bias tool was used to assess the studies. The results of systematic analysis are presented as narrative synthesis.

**Results:** Ten studies met the inclusion criteria and these full texts were reviewed. In the selected articles, the dosage of dye infusion ranged from 0.04 to 24.12 mg/kg. The effectiveness of photodynamic therapy with methylene blue against different types of cancer was confirmed by a decrease in tumor sizes in seven articles.

**Conclusion:** The results of the systematic review support the suggestions that photodynamic therapy with methylene blue helps against different types of cancer, including colorectal tumor, carcinoma, and melanoma. In cases of nanopharmaceutics use, a considerable increase of anticancer therapy effectiveness was observed. The further research into methylene blue in photodynamic anticancer therapy is needed.

**Systematic Review Registration:** (https://www.crd.york.ac.uk/prospero/display_record.php?RecordID=368738), identifier (CRD42022368738).

## 1 Introduction

**FIGURE 1:**
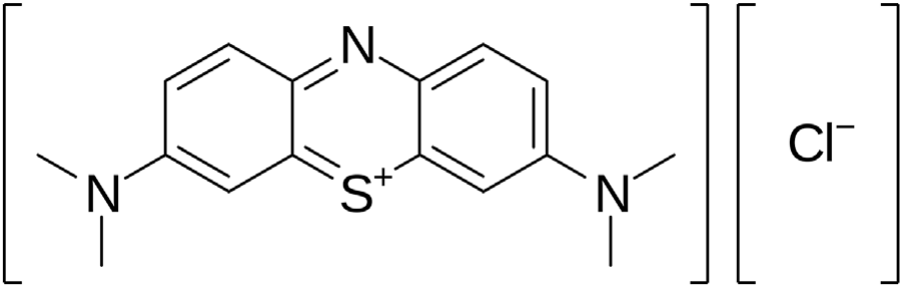
Molecular structure of MB.

Effective, safe, and low-cost anticancer compounds continue to be widely searched for and investigated, and they appear especially relevant today. In spite of the fact that biological medicinal products are in focus of pharmaceutical industry nowadays, the small molecules are continuing to be actual for clinical practice (Avdeeva et al., 2015). Drug discovery is a long, expensive, and labor-intensive process, that, however, may be optimized due to modern computational methods (Mandal and Mandal, 2009; Taldaev et al., 2022). Therefore, the re-examination of the pharmacological potential of well-known compounds is a promising focus of drug development. Methylene blue (MB)—methylthioninium chloride—can be considered as one such substance (Figure 1).

This compound was firstly synthesized as a textile dyestuff by Caro in 1876 (Friedlaender, 1877). Later, Ehrlich in cooperation with coauthors described the ability of MB to stain the nervous tissue (Ehrlich, 1886) and to act as an analgesic (Ehrlich and Leppmann, 1890) and antimalarial (Kaufmann, 1919) component. Although the clinical use of this dye was canceled due to the blue colorization of urine, it was used in malaria management throughout the 19th century (Schirmer et al., 2003). Nowadays, in the United States and the European Union, MB is applied in methemoglobinemia treatment and for staining of colorectal tumors.

Oncology seems to be a promising area for MB use thanks to its pronounced photosensitizing action that results in the disruption of pathological cells under the influence of light (Cecatto et al., 2020). This effect occurs because of the phenothiazine chromophore. It absorbs the light in the range of wavelengths from 630 to 680 nm, which leads to the generation of reactive oxygen species and the following cell death (Lim, 2021). Furthermore, MB selectively accumulates in cancer cells. These properties may be used in photodynamic anticancer therapy.

There are several clinical guidelines for photodynamic anticancer therapy, including the management of skin, pulmonary, esophageal, and cervical cancer (Yoo and Ha, 2012). However, MB is not treated as an active pharmaceutical ingredient in these guidelines. At the same time, to date, the anticancer properties of MB has received attention in the research literature. Surprisingly, systematic understanding of how effective photodynamic therapy with MB is against different types of cancer is still lacking.

Therefore, the aim of this systematic review was to evaluate the efficacy of MB in anticancer photodynamic therapy in animal models of different oncological diseases.

## 2 Methods

### 2.1 Protocol

The following systematic review was performed in accordance with the Preferred Reporting Items for Systematic Reviews and Meta-Analyses (PRISMA) guidelines (Page et al., 2021). The protocol was registered in the International Prospective Register of Systematic Reviews (PROSPERO) database in November 2022 (CRD42022368738) (Shamseer et al., 2015). Patients or public partners were not involved in the design, conduct, or interpretation of this systematic review.

### 2.2 Search strategy

To perform the electronic literature search, the PubMed database was used. The following terms were applied: {[(“methylene blue”) AND (cancer OR oncology OR antitumor)] AND (photodynamic)} NOT (antibiotic OR antimicrobial OR viruses). Any date limiters were not used.

### 2.3 Data processing

Two reviewers (IN and MK) independently and simultaneously performed an initial search and screening of articles by reading their tittles and abstracts to form the reference list. In case of disagreements, they were resolved by another author (VK). The overall inclusion and exclusion criteria that were used during screening are presented in Table 1.

**TABLE 1:** Inclusion and exclusion criteria for the systematic review.

Criteria	Inclusion	Exclusion
Animals	Adult female mice with the model of any human cancer	Use of any other species, except mice Use of young cohort (4 weeks or younger)
Intervention	Use of MB in anticancer photodynamic therapy in any formulation	Use of chemically modified MB Use of mixtures containing several active pharmaceutical ingredients except MB
Outcome measure(s)	The data on tumor size change, such as volume or area	No data on tumor size
Control	Use of untreated control or saline/PBS control	Use of light radiation without bioactive substance or with any other active ingredient
Study design	Controlled studies with separate treatment groups	Study of any other design
Language	English or Russian	Any other language

Then, two authors (RT and EM) performed the data extraction of main texts, tables, figures, and supplementary materials from the selected articles. In case of a disagreement with the inclusion criteria, the reference was excluded from the further research. The following data were the focus of the reviewers: the number of animals in experimental groups, human disease model, dose, timing, formulation, treatment method, and size of tumor. The sum of extracted outcomes was placed in Google Drive. A complete consensus in the accumulated data was reached without further disagreements.

Finally, two review authors (IR and AS) independently assessed the risk of bias using the Systematic Review Center for Laboratory Animal Experimentation (SYRCLE’s) risk of bias tool (Hooijmans et al., 2014). In case of discrepancies, they were resolved by a tiebreaker reviewer (VL).

The result of the systematic analysis is presented as narrative synthesis.

## 3 Results

### 3.1 Process of collection and selection of the studies

**FIGURE 2:**
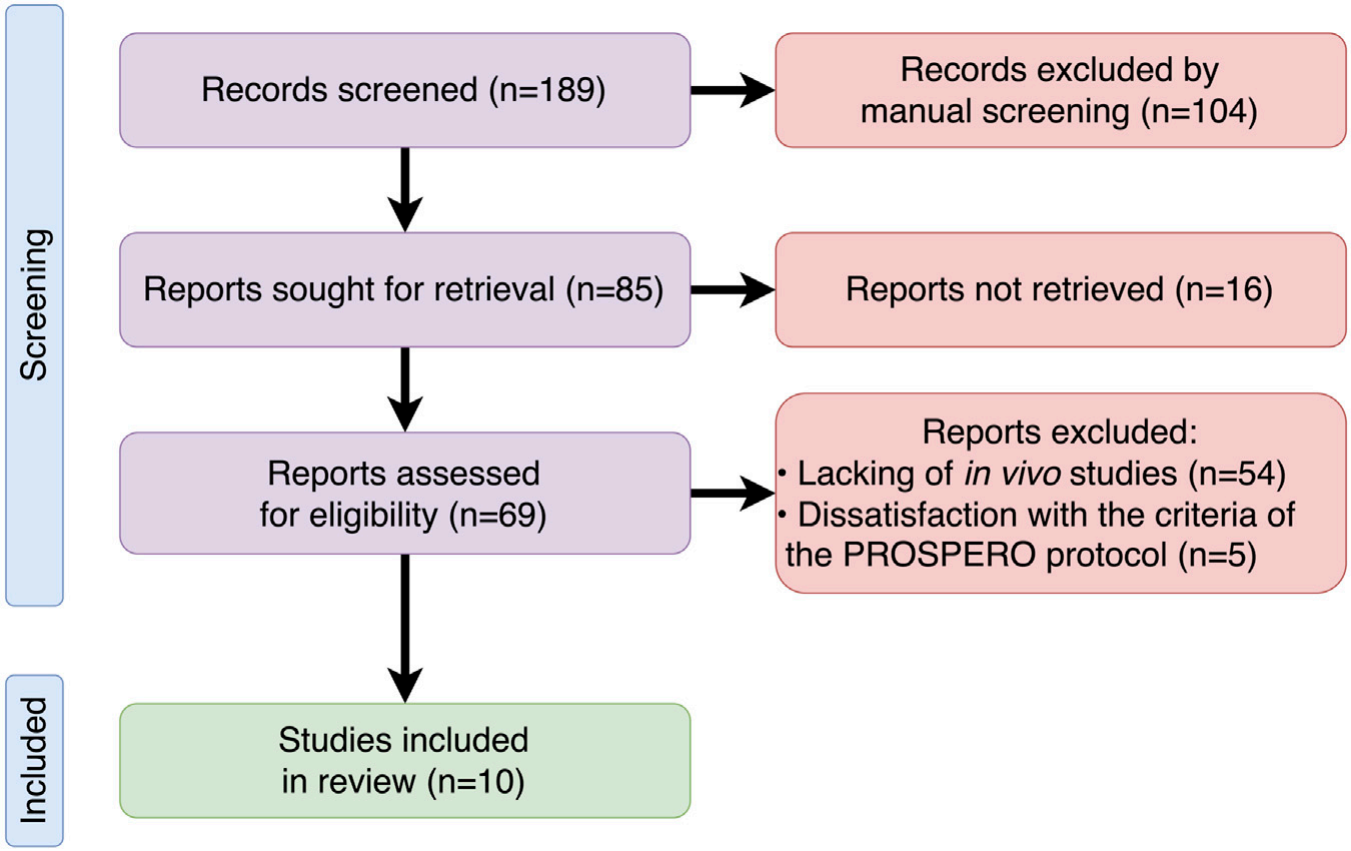
PRISMA flowchart of the search and selection process of the articles.

The initial results of the search identified 189 articles in PubMed. After the first screening, 104 articles did not meet the inclusion criteria based on their titles and abstracts, and thus they were excluded. During the further review of 85 records, an additional 75 articles were excluded due to the following reasons: the absence of access to the full text (16), lack of *in vivo* studies (54), and not meeting the criteria of the PROSPERO protocol (5). Ten total studies were included in this systematic review. The collection and selection process is illustrated in the PRISMA flow diagram (Figure 2).

### 3.2 Qualitative synthesis

The countries of origin of the studies included in the review were Brazil (3), China (4), Germany (2), and South Korea (1).

A total of 133 mice were analyzed between the 10 included studies. Fifty-nine rats were assigned to the groups administrated photodynamic cancer therapy with MB, while 74 mice served as control groups treated with placebos. These animals were used to model the different types of tumors. The majority of mice (56 individuals) were used to model colorectal cancer (Orth et al., 1998; Orth et al., 2000). Also, breast cancer was a focus of scientists and analyzed in 22 mice (Liu et al., 2017; Dos Santos et al., 2018; Xu et al., 2022). The efficacy of MB treatment against different types of skin cancer was assessed in 31 mice (Wagner et al., 2012; Silva et al., 2018). Sixteen mice were used for the assessment of the effectiveness of photodynamic cancer therapy with MB against carcinoma (Lee et al., 2015; Feng et al., 2022). The HeLa model was used in the study of eight mice (Jia et al., 2017).

All studies used injection as the intervention method. In 5 of the 10 studies, pharmaceutics were based on nanotechnologies, namely, nano-graphene oxide (Dos Santos et al., 2018), ovalbumin/polypyrrole nanoparticles (Xu et al., 2022), liposomes (Liu et al., 2017), colloidal nanoformulation (Lee et al., 2015), and nanosheet suspension (Jia et al., 2017). The MB doses were based on the individual weights of animals and varied from 0.04 to 24.12 mg/kg. At the same time, the number of intakes ranged from one injection in three studies (Orth et al., 1998; Silva et al., 2018; Xu et al., 2022) to seven injections in the study by Jia et al. (2017).

The finding from the 10 articles are summarized in Table 2. All-in-all, the majority of the studies reported on the effectiveness of photodynamic therapy with MB against different types of cancer. It was confirmed by decreases in tumor sizes reported in seven articles from 12.0% to 100.0%. Even though the treatment did not show tumor reduction in some cases of breast cancer models and the HeLa model, the application of photodynamic cancer therapy was associated with a slower tumor growth compared with the control groups.

**TABLE 2:** Summary of the findings from included studies.

Study	Disease model	Sample size		Intervention				Change of tumor size, %	
		MB	Control	Dosage form	Number of intakes	Dose, mg/kg	Length of treatment, days	MB	Control
[20]	Non-melanoma skin cancer	15	6	Injection	1	-	15	−12.0	+4.0
[17]	Breast cancer	5	5	Injection nano graphene oxide	3	2.50	12	+25.5	+48.1
[21]	Malignant melanoma	5	5	Injection	2	2.00	17	−99.0	+3060.0
[18]	Breast cancer	-	-	Injection of ovalbumin/polypyrrole nanoparticles	1	-	22	−100.0	+1100.0
[16]	Colorectal tumor	8	8	Injection	2	0.04	35	−99.9	+900.0
[19]	Breast cancer	6	6	Injection of liposome nanoplatform	3	0.75	21	+0.6	+24.9
[22]	Adenocarcinoma	3	3	Injection of colloidal nanoformulation	5	24.12	14	−60.0	+200.0
[24]	HeLa model	4	4	Injection of nanosheets suspension	7	0.80	18	+50.0	+329.4
[23]	Carcinoma	5	5	Injection	3	2.50	8	−73.0	+340.0
[15]	Colorectal tumor	8	32	Injection	1	0.40	35	−99.0	+900.0

### 3.3 Risk of bias assessment

Possible forms of bias were assessed according to the SYRCLE’s risk of bias tool.

In none of the included studies was allocation concealment during the enrolment and random housing during the experiment mentioned. Also, there was a lack of information about incomplete outcome addressing and freeing from selective outcome reporting. Moreover, the blinding of investigators during study was absent and the outcomes assessors as well as the animal selections for outcome assessment were not random. Overall, these factors resulted in the increase of performance, detection, and reporting biases.

However, dos Santos et al., Jia et al., and Feng et al. reported randomization before the allocation in analysis groups. Also, all studies except Xu et al. and Liu et al. demonstrated the groups’ similarity at the baseline. Therefore, the risk of selection bias may be considered as acceptable at least for three included articles. All studies were approved by ethical committees and followed the international guidelines for animal experiments. Thus, any conflict of interests is excluded.

**FIGURE 3:**
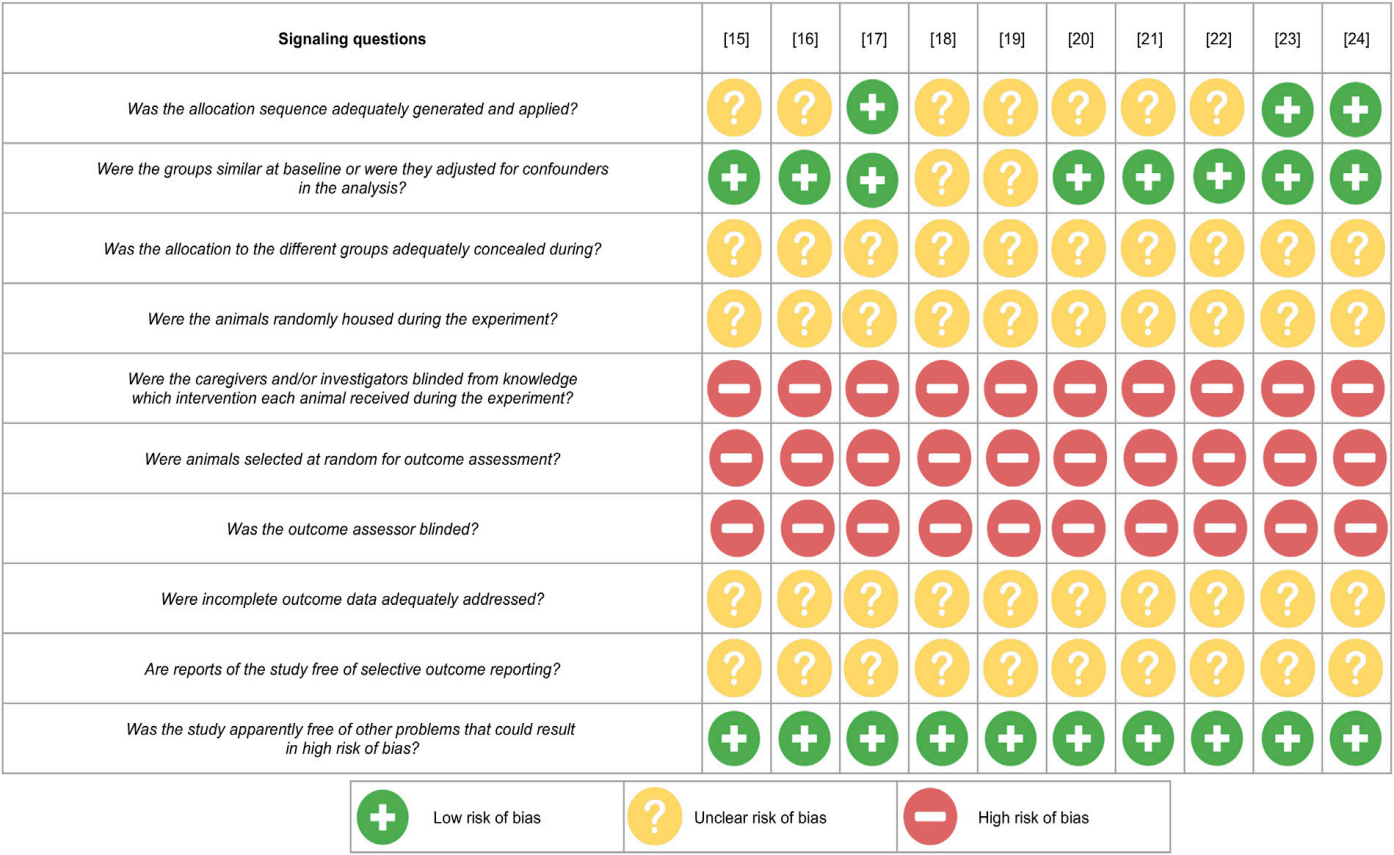
Risk of bias summary.

A summary of the low, high, or unclear risk of bias assessment of the included studies via signaling questions is presented in Figure 3.

## 4 Discussion

An initial objective of this systematic review was to identify the tendency of MB application in anticancer photodynamic therapy during preclinical studies.

As mentioned in the introduction, MB has a long history of medical application. The inexpensiveness of this compound and its wide range of biological effects may contribute to different treatment methods (Seitkazina et al., 2022). However, the baseline study showed a low affinity of MB to cancer cells (Kofler et al., 2018). Also, this dye is highly lipophilic and able to effectively cross the blood–brain barrier (Rojas et al., 2012). Furthermore, the pharmacokinetics and biotransformation of MB are complex and require detailed *in vivo* examination before its translation to clinical practice. It is therefore likely that skepticism exists for the application of MB in anticancer therapy. In contrast, Qin et al. (2011) demonstrated the increase of MB affinity when nanotechnologies are used. The conflicting results of previous studies indicate the importance of our systematic review that focused on MB in anticancer photodynamic therapy.

The potential of this treatment method was investigated in several studies in *ex vivo* models (Sahu et al., 2013; Moghassemi et al., 2022) that confirmed the cytotoxicity of the objective dye in tumor cells. Furthermore, Samy et al. (2015) demonstrated a complete response in 55% of patients with basal cell carcinoma during six sessions of photodynamic therapy with MB. At the same time, Matsubara et al. (2008) reported that MB did not inhibit osteosarcoma growth in mice. In spite of the conflicting data, the results of our systematic review show the pronounced efficacy of MB in anticancer photodynamic therapy against colorectal tumor, carcinoma, and melanoma. Also, it may inhibit the development of breast cancer since during the photodynamic therapy with MB the tumor growth was significantly lower than in control groups in several preclinical studies. The reasons of the different efficacies of treatment in various types of cancer are not clear since the mechanism of action did not change. It could conceivably be hypothesized that the bioavailability of MB in different target tissues is not equal and this results in different intensities of pharmacological effect.

The studies used innovative nanopharmaceutics to optimize the bioavailability of MB. One interesting finding of our systematic review is the suggestion that implementation of novel pharmaceutics increased the efficacy of photodynamic anticancer therapy against breast cancer even though the MB dose in nanoformulation was lower and the number of intakes did not differ. However, in carcinoma, the benefits of nanopharmaceutic were not obvious. Also, it is important to take into account the high absolute bioavailability of MB in oral formulation reported in the literature (Walter-Sack et al., 2009). Therefore, another possible explanation of treatment response variability in the different types of cancer is an unequal oxidant tolerance (Escoll et al., 2020).

The strength of present systematic review is that it included studies published in peer-reviewed journals, and the analyzed mice population was quite big. However, this research has several limitations. The included studies were characterized by heterogeneity in sample size, pharmaceutics, dosing strategies, and methods of outcome measurements. Moreover, based on the risk of bias assessment, we found poor and not methodologically flawless research supporting the efficacy of photodynamic anticancer therapy with MB. Only in three papers were the actions that prevented selection bias reported, and all studies were conducted with a high risk of detection and reporting biases. Nevertheless, taking together these data and the high safety profile of MB (Wainwright et al., 2017; Wu et al., 2018), photodynamic therapy may be considered as a promising treatment in several types of cancer.

Despite the differences observed in the selected articles, some valuable trends can be identified and can provide direction for future studies into photodynamic anticancer therapy. This work highlights the need to deepen the investigation of MB pharmacology against clinically relevant cancer models to define the rational basis for further translation. A good methodological design and the interesting pharmacokinetic characterization with modern tools will provide an opportunity to generalize these findings in meta-analyses.

## 5 Conclusion

In general, the observed results of the systematic review supported our suggestions that photodynamic therapy with MB helps against different types of cancer. Despite a modest decrease in tumor size in breast cancer and HeLa models, the results of colorectal tumor, carcinoma, and melanoma treatment were promising. We observed a considerable increase of anticancer therapy effectiveness in the cases of nanopharmaceutics use. The obtained findings inspiring us to continue the pharmaceutical development of a dosage form based on MB and the study of its therapeutic window.

## Data Availability

The original contributions presented in the study are included in the article/Supplementary Material, further inquiries can be directed to the corresponding author.
